# Development of a video-observation method for examining doctors’ clinical and interpersonal skills in a hospital outpatient clinic in Ibadan, Oyo State, Nigeria

**DOI:** 10.1186/s12913-021-06491-4

**Published:** 2021-05-22

**Authors:** Navneet Aujla, Temitope Ilori, Achiaka Irabor, Abimbola Obimakinde, Eme Owoaje, Olufunke Fayehun, Motunrayo M. Ajisola, Sinmisola O. Bolaji, Samuel I. Watson, Timothy P. Hofer, Akinyinka Omigbodun, Richard J. Lilford

**Affiliations:** 1 Warwick Medical School, University of Warwick, C/O Room B147a, CV4 7AL Coventry, United Kingdom; 2 University College Hospital, Ibadan, Nigeria; 3 University of Ibadan, Ibadan, Nigeria; 4 Institute of Applied Health Research, University of Birmingham, Birmingham, United Kingdom; 5 Department of Medicine, University of Michigan Medical School, Ann Arbor, MI USA

**Keywords:** Quality of healthcare, consultation quality, low-and middle-income countries, ambulatory care, physicians, video-observation

## Abstract

**Background:**

Improving the quality of primary healthcare provision is a key goal in low-and middle-income countries (LMICs). However, to develop effective quality improvement interventions, we first need to be able to accurately measure the quality of care. The methods most commonly used to measure the technical quality of care all have some key limitations in LMICs settings. Video-observation is appealing but has not yet been used in this context. We examine preliminary feasibility and acceptability of video-observation for assessing physician quality in a hospital outpatients’ department in Nigeria. We also develop measurement procedures and examine measurement characteristics.

**Methods:**

Cross-sectional study at a large tertiary care hospital in Ibadan, Nigeria. Consecutive physician-patient consultations with adults and children under five seeking outpatient care were video-recorded. We also conducted brief interviews with participating physicians to gain feedback on our approach. Video-recordings were double-coded by two medically trained researchers, independent of the study team and each other, using an explicit checklist of key processes of care that we developed, from which we derived a process quality score. We also elicited a global quality rating from reviewers.

**Results:**

We analysed 142 physician-patient consultations. The median process score given by both coders was 100 %. The modal overall rating category was ‘above standard’ (or 4 on a scale of 1–5). Coders agreed on which rating to assign only 44 % of the time (weighted Cohen’s kappa = 0.26). We found in three-level hierarchical modelling that the majority of variance in process scores was explained by coder disagreement. A very high correlation of 0.90 was found between the global quality rating and process quality score across all encounters. Participating physicians liked our approach, despite initial reservations about being observed.

**Conclusions:**

Video-observation is feasible and acceptable in this setting, and the quality of consultations was high. However, we found that rater agreement is low but comparable to other modalities that involve expert clinician judgements about quality of care including in-person direct observation and case note review. We suggest ways to improve scoring consistency including careful rater selection and improved design of the measurement procedure for the process score.

**Supplementary Information:**

The online version contains supplementary material available at 10.1186/s12913-021-06491-4.

## Background

Improving the quality of primary healthcare provision is now an imperative for low-and middle-income countries (LMICs) [1–6]. Recently published reports suggest that health systems in LMICs face several challenges, including workforce shortages, and limited supplies of medication and necessary medical equipment [6–8]. There are also problems with the quality of care delivered in individual healthcare encounters in LMICs, including incorrect diagnoses; poor adherence to clinical guidelines (< 50 % on average); medication errors; and provision of inappropriate care, such as unnecessary surgical interventions [6]. In addition, users express concerns about a lack of compassionate and respectful care, and low empathy [6].

In order to develop effective interventions to improve the quality of care, we first need to be able to accurately measure quality in order to better understand where any problems lie and to evaluate the effects of an intervention [9]. This paper focuses on the ‘process’ component of quality of care [10], which describes the clinical processes involving patients, such as the action of ordering a test or conducting an examination when necessary [11]. Donabedian [12] referred to this as the technical quality of care.

The most commonly used measurement methods for technical quality of care all have some key limitations in LMICs settings. Medical record documentation is sparse [13–15], in-person methods can be expensive and cumbersome to arrange [16] and, as Miller [17] suggests, testing providers may not measure practical performance in daily clinical practice. There is also the problem of the Hawthorne effect, which describes a change in behaviour as a result of being observed [18], and is evident when an observer is physically stationed in the consultation room [19]. Video-observation has a number of appealing features and offers an attractive combination of a comprehensive capture of events during an encounter, relatively low impact on the efficiency of routine healthcare delivery, and the ability to carry out the assignment of raters to encounters in a flexible and efficient way. We describe preliminary feasibility and acceptability data on the use of video in assessing the quality of clinical encounters in a LMIC setting. We also develop a checklist to evaluate consultation quality and report on the measurement characteristics of this tool. Measurement checklists that have been used to evaluate key general and symptom-specific processes of care in existing studies in LMICs tend to focus on child illnesses and have been designed for in-person observation [20–24], so are often too long and impractical to use to efficiently code video-observed consultations.

We tested our approach in the outpatient department of a large tertiary care teaching hospital in Nigeria, based on our existing work on the NIHR Global Health Research Unit on Improving Health in Slums project (https://warwick.ac.uk/fac/sci/med/about/centres/cahrd/slums/). The Improving Health in Slums project examines healthcare access and use by slum dwellers across multiple sites in South-Asia and Sub-Saharan Africa. We have evidence that a substantial proportion of people in cities seek doctor and nurse outpatient consultations at outpatient departments in hospitals. The technical quality of care provision by individual providers in the community (such as pharmacies and single-handed practices) in LMICs is reported to be poor in many studies [6, 25–30], with evidence of practices such as prescribing antibiotics for unstable angina [31]. However, only a few studies have been carried out to assess the technical quality of care in a hospital outpatient setting in LMICs [32–37]. These mostly provide only patients’ views and to our knowledge, none have used video-observation to measure the quality of care, despite the promise of the approach.

Therefore, the aim of our study is to conduct a preliminary examination of feasibility and acceptability of video-observation for assessing technical quality of care provision by doctors in a hospital outpatients’ department in a LMIC setting. A further aim is to develop measurement procedures and gain information about measurement characteristics, including validity and reliability of the measurement. Our goal was to prepare the ground for our future work on the assessment of quality of care in LMICs and to identify how we can further develop and refine our approach and measurement procedures.

## Methods

### Design and setting

We conducted a cross-sectional study in the general outpatient department of University College Hospital (UCH) in Ibadan, Oyo State, Nigeria. The UCH is a 1,000 bed teaching hospital located in south-western Nigeria. The general outpatient clinic of the hospital is the entry point for most patients presenting to UCH for primary health care services. The services are provided by consultant family physicians and supervised family physicians in training.

The study involved undertaking video-recorded observations of consecutive physician-patient consultations, followed by brief semi-structured interviews with participating physicians to understand their perceptions and experience of being video-taped during the consultations.

### Participants and eligibility criteria

The unit of observation was a single encounter with an adult patient (or child and parent/caregiver). Participating physicians were resident doctors consulting at the clinic at the time of the study who provided written informed consent to take part. Adult (> 18 years) and child patients (under 5 years) consulting with a participating physician were then also eligible to take part. Written informed consent was sought from adult patients. For child patients, written consent was provided by parents/caregivers. We did not exclude participants on the basis of presenting complaint. However, patients were only eligible if they were consulting for a new problem. Patients that did not live within Ibadan City – where UCH is situated – were excluded since our focus was care for local residents.

### Sampling and recruitment

Eligible physicians and patients were sampled opportunistically from the triage clinic, based on attendance on the day of each clinic. An average clinic week runs from Monday to Friday with two clinics (morning, 8am-1pm and afternoon, 2pm-6pm) each day. We were present in the clinic for all sessions over 10 consecutive working days from 1st to 12th April 2019. Eligible patients were identified and initially approached by a clinic coordinator. Two local project researchers were stationed at the clinic to recruit and consent individuals interested in taking part. Yoruba translated versions of the participant information leaflets and consent forms were available to patients as necessary. Ethical approval was granted by the University of Warwick Biomedical and Scientific Research Ethics Committee (REGO-2018-2306) and the University of Ibadan/UCH, Ibadan Research Ethics Committee (UI/EC/18/0646).

### Sample size

Between 60 and 70 adults and children under 5 are estimated to present on a regular clinic day at the Family Medicine Department at UCH. The number of consultations we could examine was limited by resources, but we aimed to recruit at least 120. A sample of this size would provide precision enough to estimate the mean percentage quality score in the population with a 95 % confidence interval of ± 3.7 % points at a maximum (at a value of 50 %).

### Procedure

The video-cameras were stationed in two designated consultation rooms in close proximity to the clinic’s waiting area. Two physicians were video-recorded simultaneously. Each video-camera was carefully positioned to ensure an unobstructed view of the physician. We ensured that the patient’s face was not in view and as little as possible of the back of their head was captured on the video-recording. The video-cameras were managed by the study researchers and a technician who helped to manually start and stop the recording as a patient entered and left the consultation room. Our study procedures were first piloted in the clinic. The main sample of video-recordings were later double-coded by two medically trained researchers, independent of the study team and each other, using a specially designed checklist – details for which are provided below. The coders were trained before video-coding commenced.

The study researchers carried out brief interviews with participating physicians after they had finished video-recording the full set of consultations for each physician. The brief interviews were semi-structured and facilitated through use of a topic guide that covered physicians’ reactions to being video-recorded as part of this study and potentially in future research and their prior experience of being involved in video-recorded observations (such as in medical training) (see Appendix). The interviews were conducted in-person and lasted around 10 min. A written record of the conversations was captured by the researchers in note-form. The notes were typed up, translated where necessary and securely shared as Word documents for analysis.

### Outcomes and measures

We examined four tracer symptoms: fever, cough, diarrhoea, and abdominal pain. We chose these symptoms because they are common in many LMICs, in order to enhance the generalisability of our work, and these symptoms are also red-flags for serious conditions including malaria, diarrhoea and tuberculosis. Consultations covered patients with these symptoms and patients who did not have these symptoms.

We used two approaches to measure consultation quality: an explicit checklist of key processes of care and a single global judgement-based question. Both measures examine technical and interpersonal skills including empathy. The criteria on the checklist were grouped according to the main components of the clinical encounter (interviewing/history-taking, physical examination, diagnosis and treatment, and counselling) [38], and applied to adults with specific items for child patients (see Table 1). All general criteria were applied to each consultation, and were developed based on expert feedback and adapted from criteria on checklists used in prior studies [20–24, 39]. We used these existing studies to also adapt and develop new criteria for symptom-specific clinical management alongside relevant clinical guidelines, such as the Standard Treatment Guidelines for Nigeria [40], World Health Organisation (WHO) guidelines on Integrated Management of Childhood Illnesses (IMCI) [41] and Integrated Management of Adult and Adolescent Illness (IMAI) [42]. We established a pool of explicit process measures suitable for evaluation with video-observation. The pool of criteria was subsequently reviewed by local and international clinical experts from the research team to ensure content validity and was further modified on the basis of their feedback. Raters used their own clinical judgement to guide the selection of relevant criteria in each symptom-specific checklist module.

**Table 1:** Assessment criteria, based on existing literature [20–24, 39–42] and expert feedback

General	
• Greeted patient/carer	
• Solicits what the problem is and allows patient to fully elaborate presenting problem	
• Exhibits well organised approach to information-gathering	
• Gave due attention to patient/carer (looking and listening)	
• Washed hands	
• Number of minutes spent examining patient behind the screen	
• Arranges appropriate follow-up	
• Gives patient a clear explanation of the condition, the treatment, what to look out for.	
Cough symptom	
• Asked duration of cough	
• Asked about difficulty in breathing	
• Asked about wheezing	
• Asked about presence of fever	
• Asked about sputum production	
• Asked about TB history and exposure	
• Listened to lung	
• Told to return quickly if: breathing becomes difficult, child unable to drink, child becomes more ill, child has convulsions	
Fever symptom	
• Asked about duration of fever	
• Asked about localising symptoms suggesting site of infection if not obvious (headache, neck stiffness, skin, mouth and pharynx, lungs, urinary tract, gastrointestinal tract)	
• Site of infection obvious (Yes/No)	
• If yes, examined for localising symptoms if site not obvious (neck stiffness, skin, mouth&pharynx, lungs, urinary tract, GI tract)	
• (If infant with high fever) gave paracetamol/aspirin in correct dosage	
• Advised increased fluid intake	
• Told to return in 3 days if fever persists	
Diarrhoea symptom	
• Asked duration of diarrhoea	
• Asked about presence of blood or mucus in stools	
• Asked about vomiting	
• Asked about HIV status/CD4 count	
Checked for dehydration:	
• Checked abdomen	
• Pinched skin examining for signs of severe dehydration	
• (If infant) checked for sunken fontanel	
• Treated dehydration appropriately	
• Referred case if severe or blood in stool	
• Kept child under observation if moderately dehydrated	
• Advised increased fluid intake until diarrhoea stops	
• Told how to prepare and administer oral rehydration solution	
• Told to return in 3 days if child does not improve or quickly if danger signs of dehydration appear	
Abdominal pain symptom	
• Asked about duration and progression	
• Asked about presence of fever	
• Asked about weight loss and appetite change	
• Asked about blood or mucus in stools	
• If female, asked about last menstrual period; chance of pregnancy	
• Examined abdomen for location and nature of pain, and distension	
• (If acute abdominal pain) checked for rebound tenderness • Told to return if: pain worsens, unable to tolerate liquids without vomiting, fever present	

The single global judgement-based question we used was adapted from Rubenstein [43]: Considering everything you have seen of this encounter, how would rate the overall quality of care delivered to this patient? It was developed for estimates of quality of care based on medical record review to provide an overall impression of observed quality of care in each consultation. Responses were made on a five-point Likert scale as follows: well above standard, above standard, adequate, below standard, well below standard. We presumed that this judgement-based measure would have lower reliability (or precision) than an explicit measure based on checklists. However, by virtue of allowing an expert rater to take into account a wide variety of relevant information and context apparent in the video but not captured by the explicit checklist, it has appealing strengths in terms of the validity of measurement that are distinct from but competitive with the validity conferred by the expert panels commonly used to develop explicit checklists based on guidelines. In many studies explicit and implicit judgement measures have been compared as a way to provide convergent validity for the use of both to assess quality of care and we included this measure for this purpose [44–48].

Using the rater responses, we derived two measures of quality:

- ‘Process quality score:’ the process quality score was derived using general and symptom-specific responses on the checklist. Process scores were calculated for each physician by dividing the number of positively identified criteria (numerator) by the total number of checklist criteria (general and symptom-specific) that applied for that consultation (denominator). This followed similar approaches used elsewhere (RAND Health, https://www.rand.org/health/surveys_tools/qatools.html).
- ‘Global quality rating:’ we established the global quality rating based on responses to the judgement-based question. Each physician rater completed a global quality rating for each assigned encounter.

**Fig. 1:**
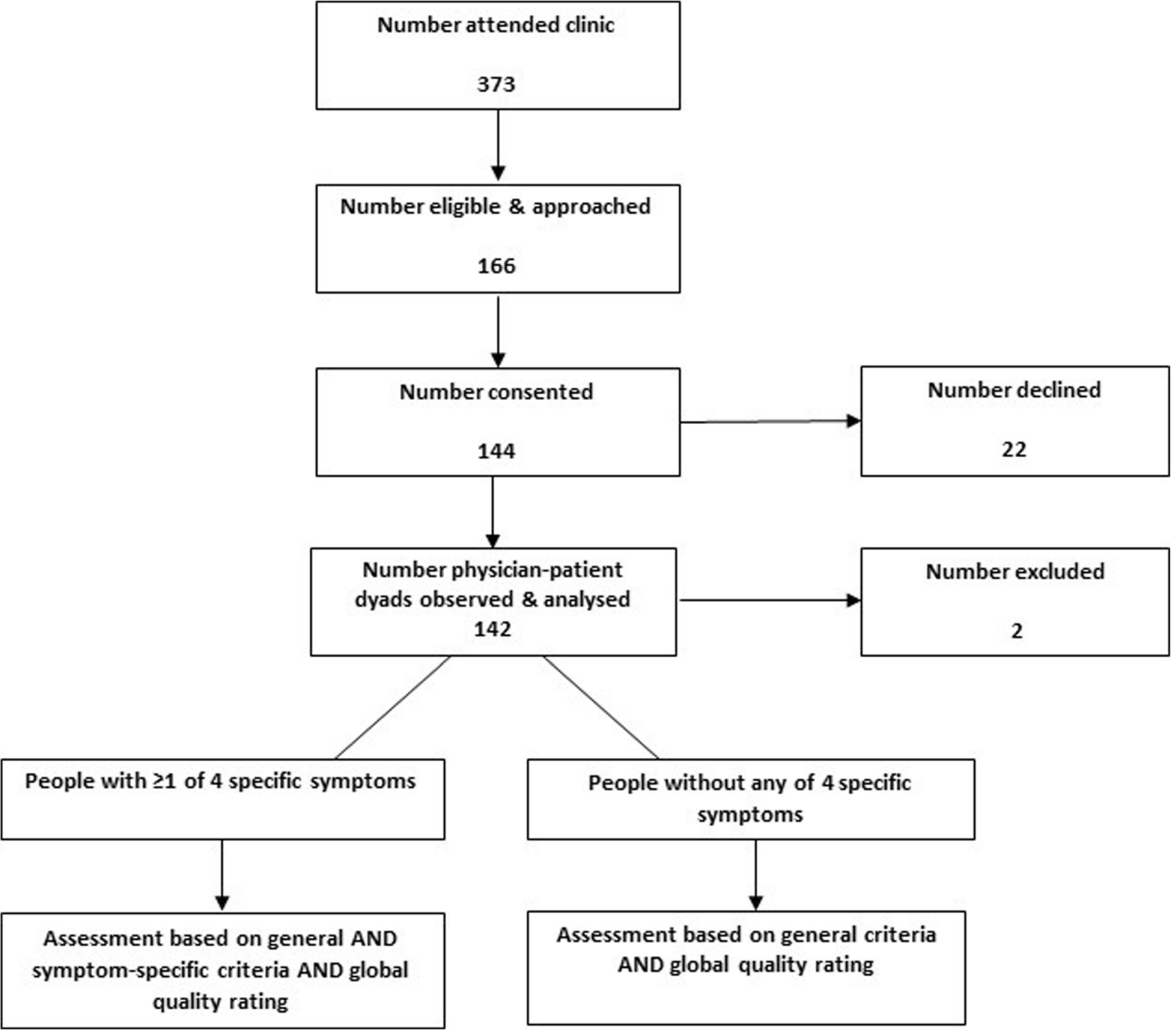
Participant flow through the main study and procedure for applying checklist criteria

Every consultation was assigned a process quality score and a global quality rating. However, process quality scores for consultations involving patients that had one or more tracer symptoms were derived from assessments of both general and symptom-specific criteria, and assessment of only the general criteria for consultations involving patients without any of the four symptoms (see Fig. 1 in the next section).

### Analysis methods

As process quality scores were not normally distributed and limited to [0 %, 100 %], we describe the median and interquartile range, and report the mode for the global quality rating. These analyses were conducted for each coder and reported separately for adult and child patients.

We examined measurement characteristics of the checklist using the following approach. For process quality scores, we estimated a Bayesian hierarchical model with three-levels including physician, patient encounters within physicians and rating occasions within patient. The model included no explanatory covariates. We estimated the proportion of total variance in quality scores that was attributable to the treating physician, differences in the ‘true’ quality of care received by one patient and the variation between rating occasions [49]. In this model, ‘true’ quality of care represents the quality score that would be obtained by an average over a very large number of rating occasions and treating physicians for an individual patient encounter. Weakly informative prior distributions were specified. The distributions used were the half t-distribution with 4 degrees of freedom (t_4_) for hierarchical standard deviation terms and the normal distribution with mean 0 and standard deviation of 5 (N(0,5^2^)) for model coefficients. These variance components were used to calculate reliability coefficients for the measurement of quality.

For the global quality rating, we describe agreement between coders visually using scatterplots, and by calculating a weighted Cohen’s kappa, which assumes a simpler model than the hierarchical model above. In order to quantify the correlation of the process score and the overall quality rating, we estimated a joint hierarchical model with the global quality rating and process quality score as outcomes, adjusting for coder effects and adult/child differences, and included a bivariate normally distributed random effect for patient in the models. Quantitative data analyses were undertaken using R version 3.4.4 and the hierarchical model was estimated using Stan 2.19.

The analysis of the brief interviews with participating physicians was guided by a thematic approach [50, 51], which was adapted to suit our data. The analysis was carried out by NA, in consultation with the interviewers (MMA and SOB).

## Results

### Participants and feasibility of the method

Nine eligible physicians were consented to the study. None of the physicians we approached declined to participate. Five out of the nine physicians were male (56 %). Most of the physicians (67 %) qualified in 2007 or after and had been practising for a median of 12-years (interquartile range (IQR): 10, 13). Figure 1 shows the flow of patient participants through the main study. Of 373 patients who attended the clinic during the recruitment period, 166 were eligible and they were invited to take part. The remainder were ineligible to take part because they resided outside of Ibadan City (> 95 %) or were involved in the piloting phase of the study. Twenty-two patients declined at this stage for several reasons including: waiting time in the clinic and a desire not to extend this time to participate in the study, a general lack of interest in taking part in the study and a preference not to have their consultation observed. Overall, 144 patients gave their consent and were observed. Two patients were excluded from the analysis because a technical issue resulted in no video-recording for these patients. We analysed 142 consultations– 112 adults and 30 children under 5 – and a process quality score and global quality rating were applied to all of these.

### Measurement characteristics of the process checklist

**Fig. 2:**
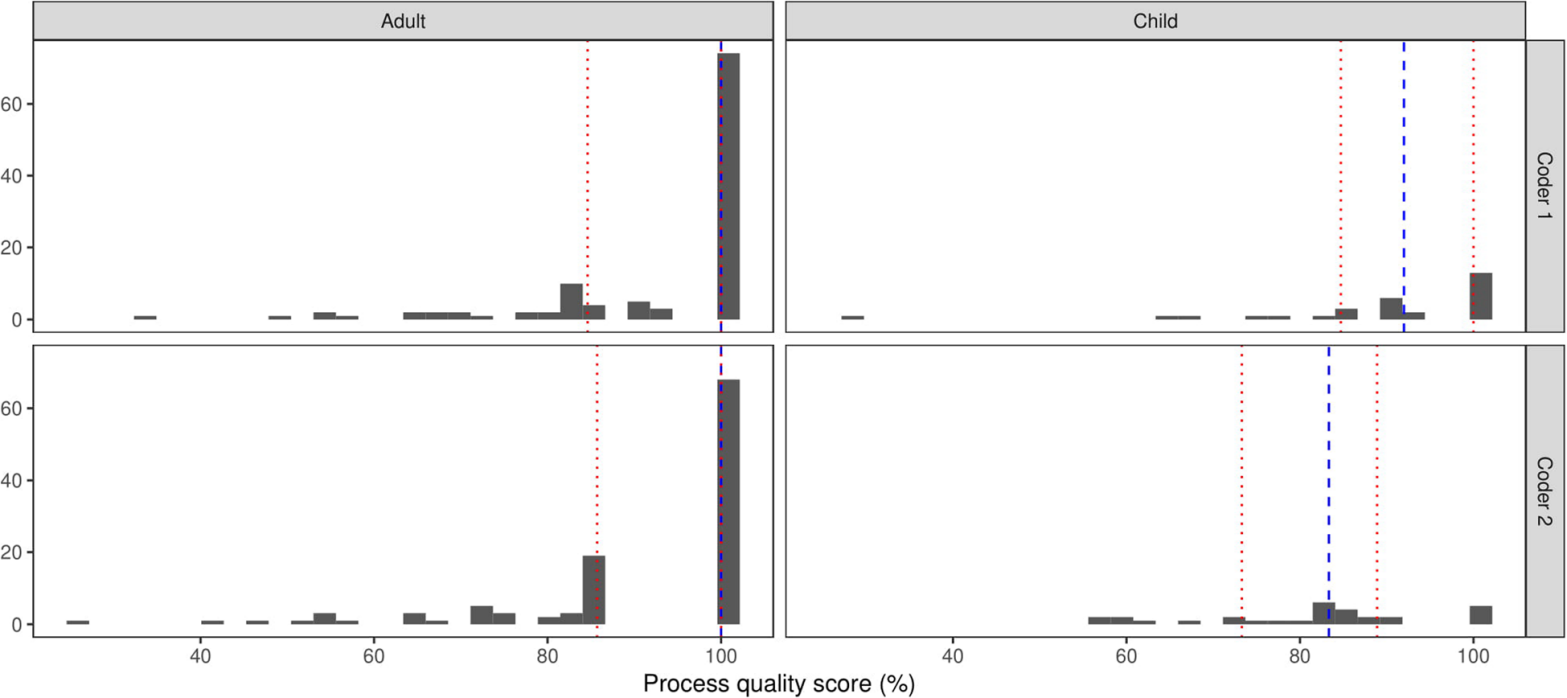
Histograms for overall process quality scores for each coder for adults versus child patients. Note: The dashed line on each figure denotes the median quality score and the dotted lines illustrates the lower and upper bounds for the inter-quartile range relating to these scores

**Fig. 3:**
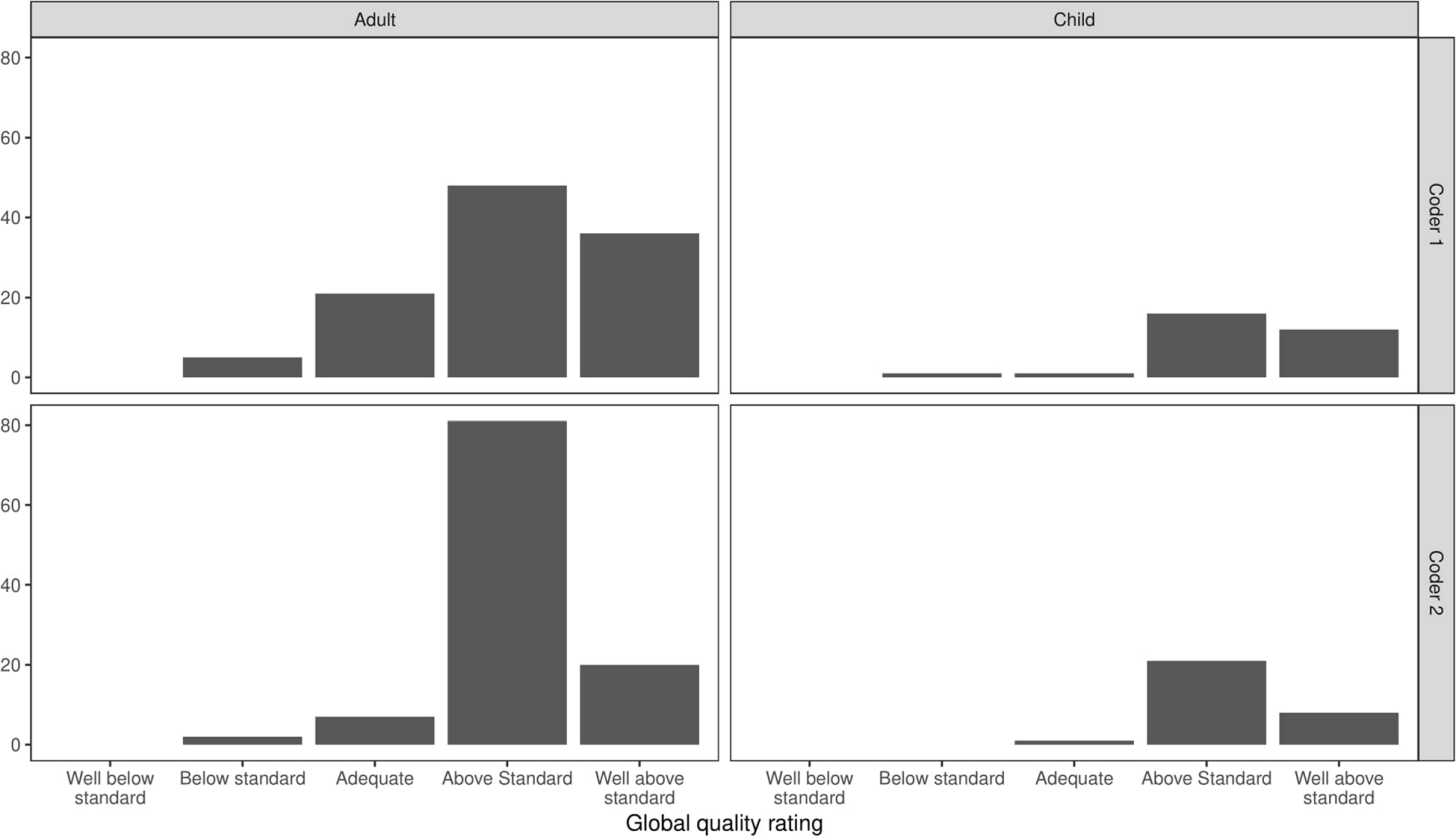
Histograms for global quality ratings for each coder for adults versus child patients

The median process quality score (calculated based on both general and symptom-specific criteria) given by both coders was 100 %. Figure 2 shows that the majority of process scores were 100 % (coder 1: IQR: 85–100 %, coder 2: IQR: 83–100 %). The modal category on the global quality judgement-based question was ‘above standard’ (see Fig. 3). Coder 1 rated 79 % of consultations as above or well above standard and 93 % for coder 2. These findings were consistent across adult and child consultations. Process quality scores were consistently high for all physicians. While the number of consultations observed varied across physicians across these consultations, seven out of the nine physicians included in the study achieved a median process score of 100 % (see Appendix Table A). The lowest score for the remainder was 83 %.

The coders agreed on process quality scores in 50 (35 %) of the 142 consultations (see Appendix Figure A). Most of the general criteria (six out of seven) were used by each coder in each consultation. For the symptom-specific modules of the checklist, coders chose how many of the symptom-specific criteria to apply in each consultation. Therefore, not all of the criteria were necessarily used for each presented symptom. Each coder included at least one criterion from each module. The overall median number of criteria used by each coder per rating occasion was six criteria. The more criteria chosen in each consultation, the worse the resulting process quality score seemed to be for the consultation (see Appendix Figure B).

The hierarchical model estimate for the process quality score was that the treating physician only accounted for 2 % of the variance. This indicates little difference in average process score between physicians. Differences in the quality of care received by patients within physician (i.e., between different consultations administered by the same physician) accounted for 22 % of the variance. The remainder of the variance was explained by variation in scores between rating occasions for the same patient encounter. For our purposes this variance component represents the noise in our measurement of quality of care. This suggests that the reliability of using the process score to distinguish between patients would be 0.24 if you selected randomly selected patients (with random providers caring for them) and measured with a single randomly selected reviewer.

### Measurement characteristics of the global quality rating

The global quality rating represents a one question summary judgement as opposed to the multi-component process score reported above. Overall, for the global quality rating, the coders agreed on which rating to assign only 44 % of the time, if exact agreement is required for ‘agreement.’ Requiring exact agreement does not make allowances for ratings that are only one category off but not exactly the same. The kappa statistic with quadratic weights gives some credit for agreement to scores that are close to each other but not exactly the same. The resulting statistic represents the reliability of the measurement for distinguishing between patients. It is comparable to an intra-class correlation reliability coefficient [52] and to the reliability calculated above for the process score. The weighted kappa was 0.26, which is described as representing ‘fair’ agreement [53].

The process score based on explicit checklists of processes of care deemed to represent good quality care and the reviewer’s implicit global judgement of quality have been used in prior literature [54] as two ways of measuring the same concept – encounter quality of care. The estimated correlation of the underlying ‘true’ quality of care (as defined above in the methods) for the two measures across all encounters in our study was very high at 0.90, as estimated with a joint hierarchical model that removes the measurement noise.

### Physicians’ views on acceptability

 We received brief feedback about our approach from all nine participating doctors. Three basic themes emerged from the data, which we briefly summarise below and indicate the number of physicians that cited each feature of each theme.

#### Performance monitoring

Most of the physicians (six out of nine) had no previous experience of being video-recorded while consulting with patients. Five out of nine reported that they liked the approach. Two out of nine explicitly stated that they would be content to be videoed again. One physician said that providing feedback to providers on their performance during the consultation should be incorporated into any performance reviews that they receive as part of their roles.

#### Awareness of the video-camera

Although around one-third of physicians said that it felt unnatural at first to be observed by the video-camera, they perceived that they found it easy to habituate and consult as they usually would. Four of the nine physicians reported that they ignored the camera from the outset of the consultation and a further two said that they forgot it was there. One physician reported that the presence of the video-camera may have encouraged them to perform better during the consultation.

#### Practical improvements to the approach

One of the physicians that reported initial self-consciousness also said that the position of the camera was too obvious. This was also reported by one of the physicians that ignored the camera. Two out of the nine physicians found it distracting for the video-camera to be manually switched on and off between consultations. Five of the nine physicians recommended inconspicuous placement of the camera so that both the doctor and patient would find it less distracting. Two participating physicians said that informed consent should be taken on a different day to video-recorded observations to help minimise their sensitisation to being observed.

## Discussion

There is a need to develop methods to accurately measure the technical quality of primary healthcare provision in a hospital outpatient department setting in LMICs. We developed a video-observation method to address this need and sought to assess the feasibility and acceptability of this mode of measurement. We were able to video-observe 142 doctor-patient consultations in the outpatients’ department of a large, tertiary care hospital (UCH) in Ibadan, Nigeria, with minimal disruption to the clinic’s daily work. Physicians were willing to participate and many told us that they liked our approach, although around a third said that they had some initial reservations about being observed.

In interviews with the physicians in our study they acknowledged that they thought about the camera suggesting the possibility of a Hawthorne effect [18], the magnitude of which we are not able to discern from our work. Prior studies of in-person direct observation in LMICs suggest that the magnitude of the Hawthorne effect from an observer in the room may be small [24] to moderate [16, 19], but declines with greater numbers of observations. Extended observation periods where possible would give participants time to desensitise to the presence of the video-camera [55]. Participants in our study also made some practical suggestions such as taking informed consent on a separate day to the observations; inconspicuous placement of the video-camera; and less intrusive alternatives to our process requiring the researcher/technician to manually start and stop the video-recording as a patient entered and left the consultation room.

One physician suggested to provide performance feedback to healthcare workers based on the observed consultation. It is possible that the opportunity to get feedback after measurement may enhance the perceived acceptability of observation to healthcare workers [56]. Further examinations of the acceptability of methods of assessing quality of care in LMICs are required [57], from the perspective of both healthcare workers and patients, including those who do and those who do not participate in studies using the approach.

An important, if familiar, issue that emerged in our study relates to the relatively low reliability or lack of precision of the measurements of quality of care. The weighted Cohen’s kappa (κ = 0.26) relating to coder agreement in assigning a single overall global quality rating to an encounter demonstrates only fair agreement. However, it is entirely consistent with the 0.2–0.4 range cited in the existing literature for implicit expert review of quality of care by experts using medical record review [58]. Given the overwhelming stability of this estimate in the literature it is unlikely that it can be improved much. At this level of reliability for a single rating, 12 independent reviews would need to be averaged to achieve a reliability of 0.8 to distinguish between individual cases [59]. The number of reviews needed to distinguish between sites of care could be more or less depending on the magnitude of the differences in quality of care between sites relative to the quality differences between patients within sites.

Given that the process quality score is based on more explicit process criteria, we had hoped that it would provide more consistent measurements with higher reliability. Yet, we observed a similar reliability of 0.24 for this measurement of quality, although reassuringly the two measures appear to be measuring the same latent construct representing quality of care as evidenced by the high adjusted correlation between the global measure and the process score. Given how much more difficult it is to curate and maintain the process quality measurement, this raises the question of whether a much simpler rating based on global judgement might be used. However, the more detailed information available from the process scores is alluring and there are several ways that this pilot work suggests that reliability of the video process scoring could be improved.

First, the overall number of criteria used was relatively small for any encounter. While the symptom probes were designed to cover common presentations, the match was clearly not ideal for the studied population. There were very few symptom-specific consultations to code overall, so more of the general criteria tended to be applied when evaluating the consultations (see Table 1). Furthermore, coders were left to independently decide when to use the symptom-specific criteria and how many were applicable to the consultation in question. In consultations where symptom-specific criteria were deemed to be relevant, coders only applied a median of one criterion. Therefore, symptom-specific coding was done using individual, rater selected criteria rather than the entire group of criteria available on the checklist, thus increasing rating occasion sources of variation. We also showed that the more criteria chosen, the worse the quality scores appeared to be. This could be a reflection of the difficulty of complex consultations and the need to prioritise tasks or an unintended consequence of the coders’ ability to choose the number of criteria to apply. It seems clear that this issue should be mitigated in future studies by two possible strategies. Focusing on a single presenting symptom would allow a single set of criteria to be used. Alternatively, we would recommend instructing coders to assign a patient specific set of criteria relevant to the presenting symptom(s) as determined by study staff or a clinical supervisor, instead of leaving the choice of criterion relevant to the consultation up to the raters.

Low reliability can result from large amounts of noise in the measurement or small differences between the targets of measurement or both [60]. Thus, a second and less appreciated reason for low reliability of quality measurement is if the observed population does not vary much in the quality of care received. For example, if encounters at outpatient academic hospitals are of consistently high quality, the reliability of a quality of care measurement procedure estimated only in that population will be lower than in a population receiving more heterogeneous levels of quality of care. We might well find that the reliability is much better when trying to distinguish quality of care received across the more varied sites of care representing the breadth of facilities where a target patient population actually receives care. Reliability is most relevant when estimated in the same population for which the measurement is intended. Thus, future studies should define the target population of people and care settings carefully.

The third important issue is the rater population. Using this measurement procedure, the true score of quality of care that we identify is the average score that would be estimated using an infinite number of raters selected from the same population from which we selected our raters. In a larger study or operational system, it would be important to carefully define the population of raters to which we wanted our scores to generalise. In our study, discrepancies could have emerged between coders relating to their differing levels of experience: one was a retired expert physician, while the other was a more junior physician. Defining the target population of raters (e.g. experts vs. senior physicians vs. community providers) is a normative decision for which there is still a lack of clarity or guidance in the existing literature [22, 55]. Our findings illustrate the need to further examine and resolve this issue.

### Strengths and limitations

To our knowledge, this is the first study to use video-observation to examine provider quality in a LMICs hospital outpatient setting, which is a particular strength of this work. Further consideration should be made to the measurement of provider quality in this setting given the dearth of literature that currently exists. A limitation of our study is the modest sample size. However, this reflects our intent to do a preliminary examination of feasibility and acceptability of video-observation for assessing technical quality of care and not to provide precise estimates of encounter quality at the patient, provider, or encounter level. The estimates of provider quality we obtained were considerably higher than those reported in prior studies in community or clinic-based primary healthcare settings in LMICs [27, 30, 31, 61–64]. This could either reflect high quality of care in the University-based practice setting we studied or be due to overly generous raters. Raters independent of the hospital and blinded to the setting will ensure that there is no “home-team” bias in scoring. In addition, we would expect more heterogeneity in scores when more diverse settings and provider types are studied and in multi-site studies. A limitation of video-observation is that similar to all forms of direct observation, video is inherently cross-sectional and cannot evaluate processes of care for an entire episode of illness, from presentation to resolution of symptoms or death. Case-note review or outcome-based measurement are methods that attempt to capture the care for an entire episode, but each have their own measurement challenges [58, 65, 66] and are particularly difficult in LMICs, where case-notes often lack sufficient detail and outcome data is not routinely collected.

### Implications and conclusion

Our study shows that video-observation is feasible and acceptable to implement in a hospital outpatients’ department in an LMICs setting, to examine the technical quality of primary care provision. However, further examinations of the acceptability of this method from the perspective of patients and providers across a broad array of settings are required. We also found that there are caveats in the use of video-observation and necessary improvements to be made to our approach. Although our study was small and preliminary, it does raise the possibility that a rating based on expert global judgement might be sufficient to assess video encounters for aggregated quality assessments at the clinic and community level and more flexible and simpler to maintain than an explicit process score. It also seems likely that low-income settings may be distinguished not just by lower overall quality but more variability in quality than high income settings. Larger scale studies across sites of care that are representative of the heterogeneity of quality of care found in a community or country are required to assess whether the procedure has sufficient reliability to practically monitor quality at the clinic or hospital level.

## Supplementary Information

**Additional file 1.**

## Data Availability

Due to the nature of this research, participants of this study did not agree for their data to be shared publicly, so supporting data is not available for this study.
