# Supplementary material for: Development of a video-observation method for examining doctors’ clinical and interpersonal skills in a hospital outpatient clinic in Ibadan, Oyo State, Nigeria
Source: BMC Health Serv Res. 2021 May 22;21:488. doi: 10.1186/s12913-021-06491-4 (PMC8141168; doi:10.1186/s12913-021-06491-4)
Supplement: Supplementary file 1 — Additional file 1. [file 12913_2021_6491_MOESM1_ESM.docx]

**APPENDIX**

**Table A: Number of observed consultations and average process quality scores across physicians**

|  | **Number of observed consultations** | **Quality score Median % (IQR)**  **CODER 1** | **Quality score Median % (IQR)**  **CODER 2** |
| --- | --- | --- | --- |
| **Physician A** | 42 | 100 (91, 100) | 86 (72, 100) |
| **Physician B** | 12 | 87 (71, 100) | 100 (83, 100) |
| **Physician C** | 13 | 100 (92, 100) | 100 (86, 100) |
| **Physician D** | 15 | 100 (0) | 86 (86, 100) |
| **Physician E** | 30 | 100 (85, 100) | 86 (79, 100) |
| **Physician F** | 10 | 92 (80, 100) | 100 (86, 100) |
| **Physician G** | 12 | 83 (60, 100) | 100 (0) |
| **Physician H** | 3 | 100 (77, 100) | 71 (71, 86) |
| **Physician I** | 5 | 100 (0) | 100 (86, 100) |

***
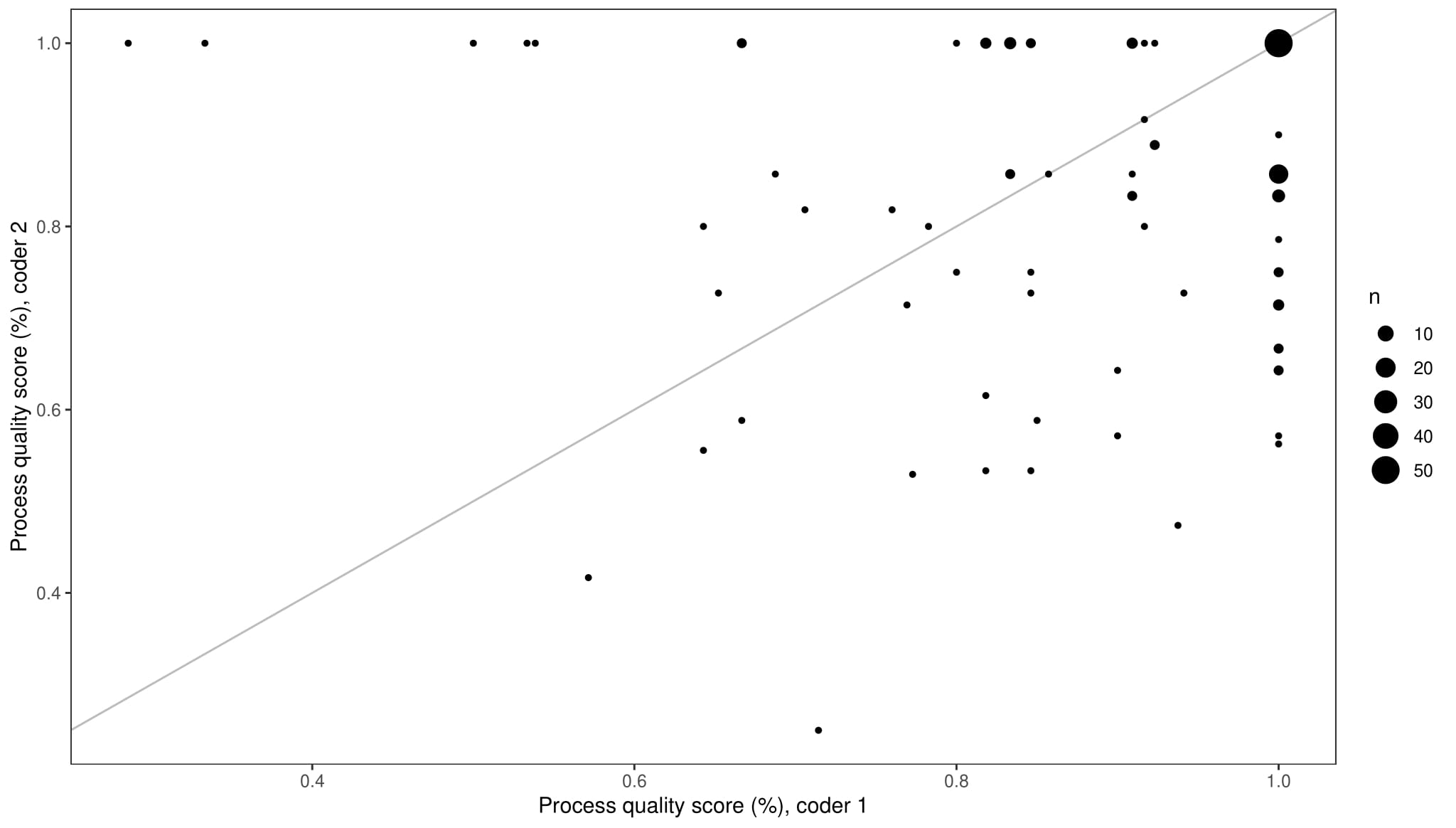
***

**Figure A: Scatterplot to illustrate agreement between coder 1 versus coder 2 process quality scores**

***
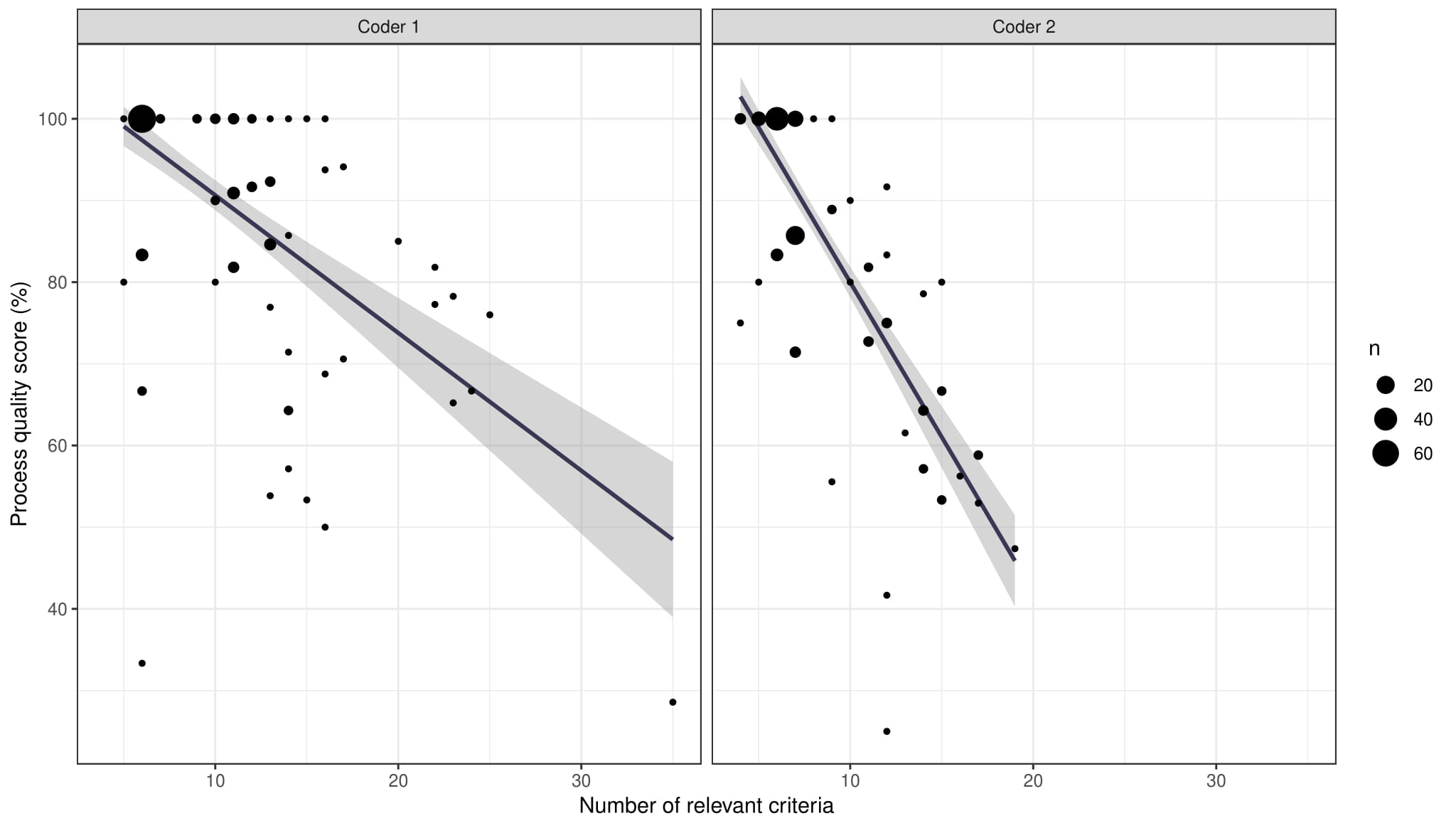
***

**Figure B. Scatterplots to illustrate the relationship between the number of relevant criteria applied and process quality scores**

**Topi guide for the brief interviews with participating physicians**

1. How did you feel about being video observed in the study?
2. What is your experience of being video observed, such as in clinical training or during practice?
3. What did you think about the positioning of the video-camera?
   *Prompt:* To what extent were you aware of the video-camera?
4. Can you suggest any improvements to our approach?
5. To what extent would you be willing to be video observed again?
6. Is there anything else that you wish to add that we have not already covered?
